# A prospective study of serum insulin-like growth factor-I (IGF-I), IGF-II, IGF-binding protein-3 and breast cancer risk

**DOI:** 10.1038/sj.bjc.6602471

**Published:** 2005-03-08

**Authors:** N E Allen, A W Roddam, D S Allen, I S Fentiman, I dos Santos Silva, J Peto, J M P Holly, T J Key

**Affiliations:** 1 Cancer Research UK Epidemiology Unit, University of Oxford, Radcliffe Infirmary, Oxford OX2 6HE, UK; 2 Academic Oncology Unit, Guy's Hospital, London SE1 9RT, UK; 3 Department of Epidemiology & Population Health, London School of Hygiene & Tropical Medicine, Keppel Street, London WC1E 7HT, UK; 4 The Institute of Cancer Research, Sutton, Surrey SM2 5NG, UK; 5 Division of Surgery, Bristol Royal Infirmary, Bristol BS2 8HW, UK

**Keywords:** breast cancer, insulin-like growth-factor-I (IGF-I), IGF-II, IGFBP-3, prospective study

## Abstract

The associations between serum concentrations of insulin-like growth factor-I (IGF-I), IGF-II and IGF-binding proteins (IGFBP)-3 and risk of breast cancer were investigated in a nested case–control study involving 117 cases (70 premenopausal and 47 postmenopausal at blood collection) and 350 matched controls within a cohort of women from the island of Guernsey, UK. Women using exogenous hormones at the time of blood collection were excluded. Premenopausal women in the top *vs* bottom third of serum IGF-I concentration had a nonsignificantly increased risk for breast cancer after adjustment for IGFBP-3 (odds ratio (OR) 1.71; 95% confidence interval (CI): 0.74–3.95; test for linear trend, *P*=0.21). Serum IGFBP-3 was associated with a reduction in risk in premenopausal women after adjustment for IGF-I (top third *vs* the bottom third: OR 0.49; 95% CI: 0.21–1.12, *P* for trend=0.07). Neither IGF-I nor IGFBP-3 was associated with risk in postmenopausal women and serum IGF-II concentration was not associated with risk in pre- or postmenopausal women. These data are compatible with the hypothesis that premenopausal women with a relatively high circulating concentration of IGF-I and low IGFBP-3 are at an increased risk of developing breast cancer.

It is well established that insulin-like growth factor-I (IGF-I) and IGF-II can stimulate cell proliferation and inhibit cell death in many tissue types (Pollak, 2000) including normal and malignant breast cancer cells (Sachdev and Yee, 2001). The bioavailability of circulating IGF ligands is complex; at least six IGF-binding proteins (IGFBPs) have been identified, the most abundant of which is IGFBP-3. This binds approximately 75–90% of circulating IGF-I and IGF-II and may be the most important determinant of IGF bioavailability (Jones and Clemmons, 1995).

There is considerable between-person variation in the circulating concentrations of IGF-I, IGF-II and their binding proteins, believed to be due to genetic and environmental factors (Jones and Clemmons, 1995; Harrela *et al*, 1996). This variation may be important because epidemiological evidence suggests that elevated levels of serum IGF-I, as absolute concentrations or relative to IGFBP-3, may be associated with an increased risk of breast cancer in premenopausal women (Peyrat *et al*, 1993; Bruning *et al*, 1995; Bohlke *et al*, 1998; Hankinson *et al*, 1998; Petridou *et al*, 2000; Toniolo *et al*, 2000; Kaaks *et al*, 2002; Krajcik *et al*, 2002; Muti *et al*, 2002; Yu *et al*, 2002; Keinan-Boker *et al*, 2003). There are limited data on the association between serum IGF-II concentration and breast cancer risk (Holdaway *et al*, 1999; Li *et al*, 2001; Yu *et al*, 2002; Grønbæk *et al*, 2004).

The aim of this study is to examine the associations between serum concentrations of IGF-I, IGF-II and IGFBP-3 and subsequent breast cancer risk in a case–control study nested within a cohort of women on the island of Guernsey.

## MATERIALS AND METHODS

### The Guernsey cohort

Between 1977 and 1991, 6127 women aged 35 years or older who lived on the British island of Guernsey were recruited into a prospective study, the main aim of which was to examine the hormonal determinants of breast cancer risk. Volunteers were sought through the local media and by using personal contacts and appeals to local women's groups. Recruitment was in two phases, from 1977 to 1985 and from 1986 to 1991; during the second phase of recruitment, women who had participated in the first phase were reinvited to the second phase (3680 women participated in both recruitment phases), and new volunteers were also sought. Study participants completed a questionnaire at interview containing details of personal characteristics and reproductive history. Height and weight measurements were taken and a nonfasting blood sample was collected. Serum was stored in 2 ml aliquots at −20°C until analysis. All participants gave voluntary written informed consent for the use of their questionnaire data and blood samples for research purposes.

Follow-up was through pathology reports (all dealt with by one pathology laboratory), Guernsey death certificates and the Wessex cancer registry. A woman was eligible for this study if she was not using any exogenous sex hormones at the time of blood collection and if she had not previously been diagnosed with cancer other than nonmelanoma skin cancer. Women who were premenopausal at recruitment were eligible if they reported that they were menstruating in their usual pattern and if their cycle length was not longer than 42 days. Postmenopausal women were eligible if they reported that they were naturally postmenopausal at the time of recruitment (defined as 1 year since their last menstrual period) or if they had undergone a hysterectomy without bilateral oophorectomy before menopause and were over 60 years old at recruitment.

Cases were women diagnosed with breast cancer up until the end of November 2000. Controls were women who were alive and free of breast cancer at the time of a case's diagnosis and who matched each case according to: age (within 2 years), date of blood collection (within 1 year) and menopausal status (pre- or postmenopausal). Cases who stated they were premenopausal at recruitment were also matched on the day of menstrual cycle (within 1 day in the category of 1–29 days until the next menstrual period and within 2 days in the category of 30+ days). Postmenopausal cases were matched to controls according to whether or not they had had a hysterectomy; and, for naturally postmenopausal cases, the number of years since menopause (1–2 years or 3+ years). Where it was not possible to match a postmenopausal case who had had a hysterectomy, controls who had been naturally postmenopausal for 3 or more years were identified. Three control subjects were randomly selected from all those who were suitably matched, except for one premenopausal case where only two controls were available. Once a control was matched to a case, she was unavailable for matching with further cases.

In total, 193 eligible breast cancer cases were identified during the follow-up period. In all, 76 cases were subsequently excluded because of insufficient sera, leaving 117 cases (70 premenopausal and 47 postmenopausal) and 350 controls for analysis (one set had two controls; the remainder had three controls).

### Measurement of serum peptide concentrations

Serum samples for all study participants were shipped on dry ice to the Division of Surgery, Bristol Royal Infirmary, UK for measurement of serum IGF-I, IGF-II and IGFBP-3. Individually matched cases and controls were analysed in the same laboratory batch and personnel were blinded as to the case–control status of samples. Double-antibody enzyme-linked immunosorbant (ELISA) assays were used to measure IGF-I (DSL-10-2800 Active; Diagnostic Systems Laboratories, Webster, TX, USA) and IGF-II (DSL-10-2600; Diagnostic Systems Laboratories). Assays for serum IGFBP-3 were carried out using a previously validated in-house double antibody radioimmunoassay (Cheetham *et al*, 1998). Total coefficients of variation (intra- and interassay combined) were determined from quality control samples randomly inserted within each batch; coefficients of variation were 6.6% for IGF-I, 12.0% for IGF-II and 3.9% for IGFBP3. Although the conditions of blood collection and the storage temperature were the same in the two phases of recruitment, the serum from the first phase had been stored longer and the tubes used for storing serum were different; therefore, we examined whether there were differences in peptide concentrations between the two phases of recruitment; among the control subjects, the medians for IGF-I, IGF-II and IGFBP3 were 21.5, 128 and 173 nmol l^−1^, respectively, for samples from the first phase of recruitment and 15.7, 131 and 159 nmol l^−1^, respectively, for samples from the second phase of recruitment. To minimise the impact of these differences on the estimates of odds ratios (OR), we categorised women into thirds based on the distribution among controls in their phase of recruitment.

### Statistical analysis

Data for pre- and postmenopausal women were analysed separately. Comparisons of selected baseline characteristics by case–control status were examined using the nonparametric Kruskal–Wallis test for continuous variables and the *χ*^2^ test for categorical variables. Age-adjusted Spearman's correlation coefficients were used to estimate the correlations between peptide hormone concentrations in controls. Serum IGF-I, IGF-II and IGFBP-3 values were categorised into thirds based on the distribution among the controls in the same phase of recruitment. The associations between serum peptide concentrations and breast cancer risk were evaluated using conditional logistic regression techniques, and relative risks estimated as ORs with their corresponding 95% confidence intervals (CIs). Adjustment for the following potential confounding factors was made, in addition to those controlled for by matching: body mass index (BMI) (<22, 22–23.9, 24–26.4, 26.5+ kg m^−2^), age at menarche (<14, 14+ years) and age at first birth (nulliparous, <24, 24–27, 28+ years). Additional adjustment for IGF-I or IGFBP-3 (included as categorical variables) was made where appropriate. The associations of the molar ratios of IGF-I/IGFBP-3 and IGF-II/IGFBP-3 with breast cancer risk were also examined. Odds ratios for each third were calculated using the lowest third as the reference category. Where appropriate, a test for linear trend was performed to assess statistical significance across exposure categories by scoring each third as 1, 2 and 3. All *P*-values were based on two-sided tests; a value of less than 0.05 was considered statistically significant. The statistical package R was used for all analyses (R Development Core Team, 2003).

## RESULTS

The median time between blood sampling and diagnosis was 13 years for both pre- and postmenopausal women; no cases were diagnosed within 3 years of recruitment. For women who were premenopausal at recruitment, the mean age at diagnosis was 57 years; we do not have information on menopausal status at diagnosis, but the majority of these cases would have been postmenopausal by the time breast cancer was diagnosed. The mean age at diagnosis among women who were postmenopausal at recruitment was 72 years.

Selected baseline characteristics of cases and controls are presented in Table 1. There were no significant differences in the median age at recruitment, age at menarche, age at first birth, age at menopause, height, weight or BMI between cases and controls among pre- or postmenopausal women. The proportions of women who were nulliparous, who had ever breast-fed, who had ever taken exogenous hormones or who were current smokers were also similar between cases and controls. The median concentrations of IGF-I, IGF-II and IGFBP-3 did not differ significantly between cases and controls in either pre- or postmenopausal women.

The correlations of the three peptide measures with each other and with other factors were examined in controls. Serum IGF-I was positively correlated with serum IGF-II (age-adjusted Spearman's rank correlation coefficient *r*=0.32 in premenopausal women and *r*=0.47 in postmenopausal women) and with IGFBP-3 (*r*=0.51 in premenopausal women and *r*=0.55 in postmenopausal women). IGF-II was also strongly positively correlated with IGFBP-3 (*r*=0.58 in premenopausal women and *r*=0.62 in postmenopausal women). IGF-I was inversely related to age, but no significant associations were found between peptide concentrations and characteristics such as BMI, smoking, parity, age at menarche, age at first birth or age at menopause in either pre- or postmenopausal women (data not shown).

Table 2 shows the risk of breast cancer associated with increasing thirds of serum IGF-I, IGF-II and IGFBP-3 in premenopausal women. Serum IGF-I concentration was not associated with breast cancer risk, either before or after adjustment for potential reproductive and other confounders; however, after statistical adjustment for serum IGFBP-3 concentration, women in the highest third of IGF-I concentration had an increased risk compared with women in the lowest third, although this was not statistically significant (OR 1.71; 95% CI: 0.74–3.95; test for linear trend; *P*=0.21). The association between the molar ratio of IGF-I/IGFBP-3 and breast cancer risk was similar to that of IGF-I adjusted for IGFBP-3 (OR 1.61; 95% CI: 0.75–3.47 in the highest *vs* the lowest third of IGF-I/IGFBP-3 concentration; test for linear trend; *P*=0.19). Serum IGFBP-3 concentration was associated with a reduction in risk, which was strengthened after statistical adjustment for BMI, age at menarche and age at first birth (OR 0.60; 95% CI: 0.29–1.24) for the highest *vs* the lowest third; test for linear trend; *P*=0.02). This association remained but was not statistically significant after additional statistical adjustment for serum IGF-I concentration (OR 0.49; 95% CI: 0.21–1.12; test for linear trend; *P*=0.07). There was no association between serum IGF-II concentration and risk in premenopausal women, either before or after statistical adjustment for IGFBP-3 (Table 2), and no association was found for the molar ratio of IGF-II/IGFBP-3 (data not shown).

For postmenopausal women, serum concentrations of IGF-I, IGF-II and IGFBP-3 were not associated with breast cancer risk either before or after statistical adjustment for potential confounding factors (Table 3).

## DISCUSSION

The findings from this small prospective study are consistent with a moderate increase in breast cancer risk associated with high levels of IGF-I, after adjustment for IGFBP-3. This is broadly consistent with previous prospective studies that have found IGF-I, as absolute concentrations or relative to IGFBP-3, to be predictive of breast cancer risk in premenopausal women (Hankinson *et al*, 1998; Toniolo *et al*, 2000; Krajcik *et al*, 2002; Muti *et al*, 2002), although one study found no association between serum IGF-I levels and risk in women who were under the age of 50 years at the time of diagnosis (Kaaks *et al*, 2002). The finding that serum IGF-I was not associated with breast cancer risk in postmenopausal women is consistent with data from other prospective studies (Hankinson *et al*, 1998; Kaaks *et al*, 2002; Krajcik *et al*, 2002; Toniolo *et al*, 2000; Keinan-Boker *et al*, 2003; Grønbæk *et al*, 2004).

Our data also show that a relatively high circulating IGFBP-3 concentration is associated with a reduction in risk in pre-, but not postmenopausal women. Although our findings are consistent with the nonsignificant inverse association reported in one previous prospective study (Hankinson *et al*, 1998), others have found either no association (Toniolo *et al* 2000; Kaaks *et al*, 2002) or an increased risk with increasing IGFBP-3 concentration in premenopausal women (Toniolo *et al*, 2000; Krajcik *et al*, 2002; Muti *et al* 2002). Nevertheless, our findings are consistent with what is currently known about the growth-inhibitory properties of IGFBP-3. It is thought that a relatively high IGFBP-3 concentration may indirectly reduce cancer risk by binding a greater proportion of circulating IGF-I, thereby reducing its bioavailability and thus inhibiting its mitogenic effects (Jones and Clemmons, 1995). In addition, IGFBP-3 has been shown to directly inhibit cell growth and induce apoptosis of breast cancer cells independently of its effect on IGF-I (Oh *et al*, 1993; Gill *et al*, 1997).

In this study, adjusting for IGFBP-3 increased the OR for the association of IGF-I with premenopausal cancers. There is considerable controversy in the literature regarding measurements of IGFBP-3. Several commentaries have noted that measurements of IGFBP-3 are still hampered by technical problems, particularly in relation to standardisation of assays. It is now evident that there is considerable heterogeneity in the forms of IGFBP-3. In addition to major differences in glycosylation and proteolytic modification, it is clear that there are many other post-translationally modified forms present in the circulation. The heterogeneity of the protein presents a considerable challenge for assay calibration. The various reports of strong and sometimes conflicting associations between IGFBP-3 concentrations and cancer risk indicate that it would be of considerable interest to measure individual forms of IGFBP-3. At present, there are no assays that can reliably measure specific characterised isoforms of IGFBP-3 and these isoforms can only really be assessed by electrophoretic techniques that are not easily applied to epidemiological studies.

Our finding of a lack of association between postmenopausal circulating levels of IGF-I and IGFBP-3 with subsequent breast cancer risk, albeit based on only 47 breast cancer cases, are also consistent with those reported by most other prospective studies, although the recent study of Grønbæk *et al* (2004) reported a positive association between IGFBP-3 and breast cancer risk. The possible difference in associations of IGF-I and IGFBP-3 and risk by menopausal status suggests that high levels of IGF-I and/or low levels of IGFBP-3 may be important in breast cancer development in younger, but not older, women. If confirmed, the stronger association of IGF-I with risk in younger than in older women might be because IGF-I levels are higher, or perhaps because oestrogen levels are high. IGF-I and IGF-II may be indirectly acting as an indicator of sex hormone activity in younger women or they may be directly interacting with oestrogens to increase breast cancer risk. Indeed, oestradiol has been shown to upregulate expression of the IGF-I receptor and both hormones can interact in a synergistic manner to stimulate breast cancer cell proliferation (Yee and Lee, 2000; Hamelers and Steenbergh, 2003). However, there are limited epidemiological data on the potential joint effect of endogenous oestrogens and IGFs on breast cancer risk (Yu *et al*, 2003), and other, as yet unknown, age-related changes might influence this association.

This is the first study to examine prospectively the association between serum IGF-II concentration and breast cancer risk in premenopausal women. Although IGF-II has a clearly established role in foetal development (Allan *et al*, 2001), its function in postnatal life is less understood, despite circulating at a much higher concentration than IGF-I. Although experimental evidence suggests that IGF-II can increase the growth of breast cancer cells *in vitro* (Cullen *et al*, 1992) and is upregulated in many breast tumours (Li *et al*, 1998; Fichera *et al*, 2000), our results show that circulating IGF-II concentration is not strongly associated with subsequent breast cancer risk in pre- or postmenopausal women, which is consistent with evidence from case–control studies (Holdaway *et al*, 1999; Li *et al*, 2001; Yu *et al*, 2002), although Grønbæk *et al* (2004) reported in a prospective study an increase in risk of breast cancer among postmenopausal women with increasing IGF-II, which was of borderline statistical significance.

The main strengths of this study are the prospective design with cases and controls matched according to predefined established risk factors for breast cancer. In addition, blood samples were taken at least 3 years before cancer diagnosis, thereby reducing the possibility that differences in IGF levels are a result of an undiagnosed breast tumour at the time of blood collection. Although peptide measurements were made in a single blood sample from each woman, previous studies suggest that this reliably reflects long-term IGF-I and IGFBP-3 levels in adult women, with intraclass correlation coefficients between 0.73 and 0.87 for samples taken a year apart (Kaaks *et al*, 2000; Borofsky *et al*, 2002), and Hunt *et al* (2002) have reported a Spearman rank correlation of 0.81 for measures of IGF-II in two samples taken at different rounds of recruitment. Further, all assay measurements were conducted blinded and in case–control sets, thereby minimising the impact of laboratory variation on case–control comparisons. However, little is known about the correlation between serum concentrations and bioactive tissue levels. Although IGFs originate mainly in the liver, they can also be produced locally in breast cancer cells and can thus act in an autocrine/paracrine manner as well as through endocrine pathways (Sachdev and Yee, 2001). Nevertheless, the observation that a reduction in circulating IGF-I can reduce tumour growth in animals (Dunn *et al*, 1997; Wu *et al*, 2003) suggests that blood levels of IGF-I are biologically meaningful and can serve as an indicator of the total pool of IGF-I available to cells (Holly and Hughes, 1994).

In summary, the results of this prospective study are consistent with the hypothesis that premenopausal women with a relatively high circulating concentration of IGF-I and low IGFBP-3 are at an increased risk of developing breast cancer.

## Figures and Tables

**Table 1:** Baseline characteristics of breast cancer cases and controls by menopausal status^a^

	**Premenopausal women**			**Postmenopausal women**		
	**Cases (*n*=70)**	**Controls (*n*=209)**	***P*-value^b^**	**Cases (*n*=47)**	**Controls (*n*=141)**	***P*-value^b^**
Age at recruitment (years)	41 (37–44)	41 (37–44)	0.83	59 (55–62)	58 (55–63)	0.98
Age at menarche (years)	13 (12–14)	13 (12–14)	0.35	14 (12–15)	13 (12–14)	0.49
Age at first birth^c^ (years)	23 (21–26)	24 (22–27)	0.62	26 (22–29)	25 (23–28)	0.58
Age at menopause (years)	N/A	N/A		50 (47–52)	50 (48–53)	0.24
Height (cm)	162 (157–165)	161 (157–165)	0.95	158 (155–164)	160 (155–163)	0.52
Weight (kg)	63 (59–69)	62 (56–68)	0.20	62 (58–70)	63 (58–69)	0.96
BMI (kg m^−2^)	24 (23–27)	24 (22–26)	0.16	25 (23–28)	25 (23–27)	0.54
						
*Parity*						
Nulliparous	6 (9%)	25 (12%)		12 (26%)	20 (14%)	
1	8 (11%)	28 (13%)		4 (9%)	28 (20%)	
2	35 (50%)	83 (40%)		14 (30%)	36 (26%)	
3+	21(30%)	73 (35%)	0.50	17 (35%)	57 (40%)	0.13
						
*Past OC use*						
Yes	54 (77%)	152 (73%)	0.47	9 (19%)	27 (19%)	1.00
						
*Past HRT use* d						
Yes	N/A	N/A	N/A	11 (24%)	33 (24%)	0.98
						
*Serum peptides*						
IGF-I (nmol l^−1^)^e^	22.4 (18.6–26.9)	22.2 (18.5–26.5)	0.88	16.4 (12.8–23.3)	16.8 (13.5–21.2)	0.86
IGF-II (nmol l^−1^)^e^	124 (107–144)	129 (114–148)	0.27	125 (111–164)	131 (116–151)	0.63
IGFBP-3 (nmol l^−1^)	170 (141–193)	172 (155–194)	0.10	167 (139–194)	164 (142–194)	0.93

OC=oral contraceptive; HRT=hormone replacement therapy; IGF=insulin-like growth factor; IGFBP=IGF-binding proteins; N/A=not applicable.

a Values are medians (interquartile range) or numbers (percent).

b *P*-value based on Kruskal–Wallis rank sum test for continuous variables and *χ*^2^ test for categorical variables.

c Among parous women only.

d Data were missing for three postmenopausal women.

e Serum IGF-I and IGF-II measurements were unavailable for one premenopausal case.

**Table 2:** ORs for breast cancer by tertiles of IGF-I, IGF-II and IGFBP-3 for premenopausal women

	**Tertile 1**	**Tertile 2**	**Tertile 3**	***P* for trend**
*IGF-I*				
Number of cases/controls^a^	22/70	22/71	25/68	
OR, crude^b^	1.00	0.96 (0.49–1.92)	1.20 (0.60–2.37)	0.61
OR, adjusted^c^	1.00	0.98 (0.49–1.99)	1.19 (0.58–2.46)	0.63
OR, adjusted^d^	1.00	1.25 (0.58–2.69)	1.71 (0.74–3.95)	0.21
				
*IGF-II*				
Number of cases/controls^a^	25/70	23/69	21/70	
OR, crude^b^	1.00	0.92 (0.48–1.77)	0.85 (0.43–1.70)	0.65
OR, adjusted^c^	1.00	1.02 (0.52–2.01)	0.86 (0.42–1.76)	0.68
OR, adjusted^d^	1.00	1.27 (0.61–2.64)	1.17 (0.48–2.84)	0.70
				
*IGFBP-3*				
Number of cases/controls	32/70	16/69	22/70	
OR, crude^b^	1.00	0.51 (0.26–1.00)	0.66 (0.33–1.31)	0.19
OR, adjusted^c^	1.00	0.44 (0.21–0.93)	0.60 (0.29–1.24)	0.02
OR, adjusted^e^	1.00	0.40 (0.19–0.86)	0.49 (0.21–1.12)	0.07

OR=odds ratios; IGF=insulin-like growth factor; IGFBP=IGF-binding proteins; BMI=body mass index.

a Serum IGF-I and IGF-II measurements were unavailable for one premenopausal case.

b Crude (matched for age at recruitment, date of blood collection, day of menstrual cycle).

c Adjusted for BMI, age at menarche and age at first birth.

d Adjusted for BMI, age at menarche, age at first birth and IGFBP-3.

e Adjusted for BMI, age at menarche, age at first birth and IGF-I.

**Table 3:** ORs for breast cancer by tertiles of IGF-I, IGF-II and IGFBP-3 for postmenopausal women

	**Tertile 1**	**Tertile 2**	**Tertile 3**	***P* for trend**
*IGF-I*				
Number of cases/controls	18/48	13/46	16/47	
OR, crude^a^	1.00	0.76 (0.34–1.70)	0.90 (0.42–1.97)	0.80
OR, adjusted^b^	1.00	0.62 (0.25–1.56)	0.77 (0.34–1.74)	0.56
OR, adjusted^c^	1.00	0.60 (0.24–1.55)	0.73 (0.29–1.84)	0.52
				
*IGF-II*				
Number of cases/controls	20/47	10/47	17/47	
OR, crude^a^	1.00	0.52 (0.22–1.21)	0.83 (0.38–1.83)	0.64
OR, adjusted^b^	1.00	0.45 (0.19–1.20)	0.87 (0.37–2.05)	0.67
OR, adjusted^c^	1.00	0.43 (0.17–1.10)	0.81 (0.29–2.28)	0.63
				
*IGFBP-3*				
Number of cases/controls	16/47	15/47	16/47	
OR, crude^a^	1.00	0.94 (0.43–2.08)	1.00 (0.43–2.34)	1.00
OR, adjusted^b^	1.00	0.99 (0.43–2.27)	0.99 (0.40–2.46)	0.98
OR, adjusted^d^	1.00	1.06 (0.45–2.47)	1.14 (0.40–3.23)	0.81

OR=odds ratios; IGF=insulin-like growth factor; IGFBP=IGF-binding proteins.

a Crude (matched for age at recruitment, date of blood collection, number of years postmenopausal or hysterectomy).

b Adjusted for BMI, age at menarche and age at first birth.

c Adjusted for BMI, age at menarche, age at first birth and IGFBP-3.

d Adjusted for BMI, age at menarche, age at first birth and IGF-I.
